# Massive non-natural proteins structure prediction using grid technologies

**DOI:** 10.1186/1471-2105-10-S6-S22

**Published:** 2009-06-16

**Authors:** Giovanni Minervini, Giuseppe Evangelista, Laura Villanova, Debora Slanzi, Davide De Lucrezia, Irene Poli, Pier Luigi Luisi, Fabio Polticelli

**Affiliations:** 1 Department of Biology, University Roma Tre, Viale G. Marconi 446, Rome, I-00146, Italy; 2 European Centre for Living Technology, Calle del Clero, S. Marco 2940, Venice, I-30124, Italy; 3 Department of Statistics, University of Padua, Via Cesare Battisti 241, Padua, I-35121, Italy; 4 Department of Statistics, University Ca' Foscari of Venice, San Giobbe, Cannaregio 873, Venice, I-30121, Italy

## Abstract

**Background:**

The number of natural proteins represents a small fraction of all the possible protein sequences and there is an enormous number of proteins never sampled by nature, the so called "never born proteins" (NBPs). A fundamental question in this regard is if the ensemble of natural proteins possesses peculiar chemical and physical properties or if it is just the product of contingency coupled to functional selection. A key feature of natural proteins is their ability to form a well defined three-dimensional structure. Thus, the structural study of NBPs can help to understand if natural protein sequences were selected for their peculiar properties or if they are just one of the possible stable and functional ensembles.

**Methods:**

The structural characterization of a huge number of random proteins cannot be approached experimentally, thus the problem has been tackled using a computational approach. A large random protein sequences library (2 × 10^4 ^sequences) was generated, discarding amino acid sequences with significant similarity to natural proteins, and the corresponding structures were predicted using Rosetta. Given the highly computational demanding problem, Rosetta was ported in grid and a user friendly job submission environment was developed within the GENIUS Grid Portal. Protein structures generated were analysed in terms of net charge, secondary structure content, surface/volume ratio, hydrophobic core composition, etc.

**Results:**

The vast majority of NBPs, according to the Rosetta model, are characterized by a compact three-dimensional structure with a high secondary structure content. Structure compactness and surface polarity are comparable to those of natural proteins, suggesting similar stability and solubility. Deviations are observed in α helix-β strands relative content and in hydrophobic core composition, as NBPs appear to be richer in helical structure and aromatic amino acids with respect to natural proteins.

**Conclusion:**

The results obtained suggest that the ability to form a compact, ordered and water-soluble structure is an intrinsic property of polypeptides. The tendency of random sequences to adopt α helical folds indicate that all-α proteins may have emerged early in pre-biotic evolution. Further, the lower percentage of aromatic residues observed in natural proteins has important evolutionary implications as far as tolerance to mutations is concerned.

## Background

A fundamental question in protein science is if the known natural proteins are just one of the many possible ensembles of stable and functional polypeptides or the only possible solution found by molecular evolution. In other words, is it possible to imagine many biochemical "parallel dimensions" or the one we know is the only possible one? This question has many implications in terms of our knowledge of the principles underlying the proteins sequence/structure/function relationships, and of our ability to modify the existing proteins, or design novel proteins, for biotechnological and biomedical purposes. In fact, the number of known natural protein sequences, though quite large, is infinitely small compared to the number of proteins theoretically possible with the twenty natural amino acids. Thus, there exists a huge number of protein sequences which have never been exploited by living organisms, named by Luisi and coworkers "never born proteins" (NBPs) [1]. Just to give an example, the latest release of UniProtKB/Swiss-Prot (56.2 of 23 September 2008) contains approx. 400 thousand sequence entries [2], many of which are evolutionary related. On the other hand, 100^20 ^chemically different proteins can be in principle obtained with the 20 natural amino acids considering random polypeptides of only 100 amino acids in length (the average length of natural proteins being 360 amino acids [2]). In this regards, a key issue is if natural protein sequences were selected during molecular evolution because they have unique physico-chemical properties or else they just represent a contingent subset of all the possible proteins with a stable and well defined fold. If the latter hypothesis were true, this would mean that the protein realm could be exploited to search for novel folds and functions of potential biotechnological and/or biomedical interest. Such a problem cannot be easily tackled experimentally as this would require the production and structural characterization of a huge number of random polypeptides. Attempts have been made in this direction [1], however an alternative is that of adopting a computational approach which, though yields only predictive results, allows to sample a much larger sequences space. In addition, a computational approach allows to evenly sample the protein sequences space in different regions far away enough from the ensemble of natural proteins.

In this work, we describe the study of the structural properties of a large library of random protein sequences with no significant similarity with natural proteins by means of the well known *ab initio *protein structure prediction software Rosetta *abinitio *[3]. Rosetta *abinitio *has consistently been shown to yield accurate, and in some cases near-atomic resolution, predictions of protein structures even in the absence of evolutionary information [4], thus representing the right tool to address the problem of NBPs structure prediction.

Results obtained indicate that most of the NBPs are characterized by three-dimensional structures comparable to those of natural proteins in terms of compactness and surface polarity. However, α helix content and aromatic/aliphatic residues ratio is significantly higher in NBPs as compared to natural proteins of comparable length. The evolutionary implications of these results are discussed.

## Methods

### Amino acid sequences library generation

Random amino acid sequences (70 amino acids long) were generated using the utility RandomBLAST whose implementation has been described in detail elsewhere [5]. Briefly, RandomBLAST consists of two main modules: a pseudo random sequence generation module and a BLAST software [6] interface module. The first module uses the Mersenne Twister 19973 pseudo-random number generation algorithm [7] to generate pseudo-random numbers between 0 and 19. which are translated in single character amino acid code and then concatenated to reach the desired sequence length. Each sequence generated is then given in input to the second RandomBLAST module, an interface to the blastall program which invokes the following command:

**blastall -m 8 -p blastp -d database -b 1**;

where **database **in our case stands for the NR database (the National Center for Biotechnological Information non redundant protein sequence database [8]), and the parameters **-m 8 **and **-b 1 **indicate the alignment format (tabular form) and the number of sequences to be returned (just the first hit), respectively. Blastall output is then retrieved by RandomBLAST and the *Evalue *extracted from it. If the *Evalue *is greater than or equals the threshold chosen by the user, the sequence is added to the output file. Note that in this case only the sequences that do not display significant similarity to any protein sequence present in the database are considered valid, so that, contrary to the normal BLAST usage, valid sequences are those displaying an *Evalue *higher than the threshold, set to a value of 1 [9]. The total number of NBPs sequences generated was 20496.

### NBPs three-dimensional structure prediction

NBPs three-dimensional structures have been predicted using Rosetta *abinitio*, an *ab initio *protein structure prediction software based on the assumption that in a polypeptide chain local interactions bias the conformation of sequence fragments, while global interactions determine the three-dimensional structure with minimal energy which is also compatible with the local biases [4,10]. To derive the local sequence-structure relationships for a given amino acid sequence (the query sequence) Rosetta *abinitio *uses the Protein Data Bank [11] to extract the distribution of conformations adopted by short segments in known structures. The latter is taken as an approximation of the distribution adopted by the query sequence segments during the folding process [10].

Given the extent of the amino acid sequences dataset under study, a large amount of computational resources were needed to accomplish the task of their structure prediction. Thus, Rosetta *abinitio *has been deployed on the EUChinaGRID grid infrastructure and a user friendly job submission environment was developed within the GENIUS Grid Portal [12-14]. A detailed description of the porting of Rosetta *abinitio *in grid can be found elsewhere [12]. Briefly, the application execution in grid was first tested using a shell script which registers the program executable (pFold.lnx) and the required input files on the grid file catalogue (LFC catalogue), calls the Rosetta *abinitio *executable and proceeds with workflow execution. A JDL (Job Description Language) file was also created to run the application on the grid working nodes which use the gLite middleware [12,15].

Once the correct execution of the program in grid was assessed, a user friendly interface was developed within GENIUS to allow users with poor knowledge of the grid middleware to submit, monitor the execution and download the output of a high number of Rosetta *abinitio *predictions in grid [12].

### Three-dimensional structures analysis

The analysis of the physico-chemical properties of the predicted protein structures was carried out using a collection of different tools. For each tool the analysis procedure was automated using *ad hoc *Perl scripts. In detail, the programs used were MSMS [16], for molecular volume calculation, SURFace Algorithms [17], for surface properties analysis (overall molecular surface, per residue solvent accessibility), Freqaa [18], for amino acid composition analysis and DSSP [19] for secondary structure content analysis. Surface hydrophobicity was calculated as the ratio between the solvent exposed surface of hydrophobic amino acids and the total solvent exposed surface, both calculated using SURFace Algorithms [17].

To compare the properties of NBPs to those of natural proteins structures, a dataset of natural proteins of length comparable to that of NBPs (55 to 95 amino acids long sequences as compared to NBPs 70 amino acids long sequences) was derived from the Protein Data Bank [11]. The dataset was cleaned up eliminating protein fragments and proteins whose fold is determined by macromolecular complexes formation. The final natural proteins dataset was formed by 866 proteins.

### Statistical analysis of the data

A first exploratory data analysis has been developed to see if there were any significant difference in the structure observed in the two data-sets. Initially few outliers in the data that could affect this analyses were removed, generating a dataset of 18465 NBPs and a dataset of 839 natural proteins. Based on the probability distributions estimated from the data, the outliers were simply the values with probability of occurrence smaller than 0.005. In order to detect in a clear and a prompt way the pattern in the data, the outliers were initially removed and their presence was not relevant in the exploratory analysis. However they were considered in the subsequent study with datasets of comparable size. For these sets measures of location, index of dispersions, correlations matrix were derived, and box-plots and scatterplots were built to compare the two data sets. This study was performed on different structure-related variables, which include: volume, surface, surface/volume ratio, net charge, secondary structure content, and surface hydrophobicity. Tests on the Gaussian distribution of the variables led to reject the hypothesis of Gaussianity for the majority of the variables investigated. With a test significance level of 0.05 almost all the variables result with statistically different mean and variance for the two data sets. The analysis has been also conducted on smaller data-sets of comparable size: a random sample of 1000 observations has been drawn from NBPs dataset and comparisons have been developed. The two analyses generated similar conclusions, presented in the following section. The statistical software used to analyse the data was R [20].

## Results

### Amino acid composition analysis

**Figure 1:**
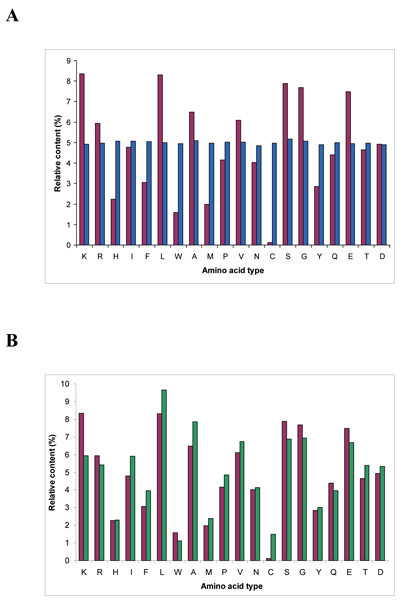
**Amino acid composition of NBPs and natural proteins**. A) Comparison between the amino acid composition of NBPs (blue bars) and that of selected natural proteins of comparable length (purple bars; see Methods). B) Comparison between the amino acid composition of the natural proteins subset used in this work (purple bars) and that of the whole UniProt dataset [2] (green bars).

Figure 1 shows the amino acid composition of natural and random protein sequences datasets. For random proteins dataset each amino acid relative content is about 5%, an obvious consequence of the random nature of these amino acid sequences, even though in individual NBPs sequences, large variations in amino acid composition are observed, as indicated by the high standard deviation calculated values (Table 1). Interesting differences are observed when random sequences composition is compared to that of natural proteins. In detail, for the natural proteins dataset five amino acids are significantly over represented: Lys (8.35%), Leu (8.30%), Ser (7.88%), Gly (7.69%) and Glu (7.48%), while three amino acids are under represented: Trp (1,58%), Met (1,98%) and Cys (0,13%) (Figure 1). These differences are not a peculiar characteristic of the subset of natural proteins chosen in this work. In fact similar amino acid composition is observed for the UniProt dataset containing all the known protein sequences [2] (Figure 1), indicating that the natural proteins subset used in this work is representative of all natural proteins, at least as far as amino acid composition is concerned. Notable differences are observed for Lys and Cys residues which display the highest and lowest percentage in the natural dataset. The former finding can be explained considering that a consistent number of natural proteins with a length in the 55–95 residues range display nucleic acid binding activity and thus a basic character. Cys residues percentage is less straightforward to explain, even though it is probably connected to the high reactivity of this amino acid whose presence in natural proteins is tightly evolutionary controlled. Turning to the random proteins dataset, these display a significant excess of aromatic amino acids and a strikingly lower content in Leu, as also evidenced by the lower ratio between aliphatic and aromatic residues as compared to the natural dataset (Table 2).

**Table 1:** Mean and Standard deviation values of amino acids relative content in the NBPs dataset.

	**Mean** **(%)**	**Std. Dev. (%)**
**Gly**	4.90	2.57
**Pro**	4.90	2.53
**Ala**	4.97	2.63
**Val**	4.92	2.62
**Leu**	5.02	2.62
**Ile**	4.90	2.57
**Met**	4.99	2.63
**Cys**	4.89	2.57
**Phe**	4.89	2.59
**Tyr**	4.96	2.59
**Trp**	4.90	2.53
**His**	4.92	2.57
**Lys**	4.93	2.59
**Arg**	4.99	2.63
**Gln**	4.92	2.59
**Asn**	4.89	2.54
**Glu**	4.96	2.62
**Asp**	4.92	2.59
**Ser**	4.93	2.62
**Thr**	4.96	2.57

**Table 2:** Hydrophobic amino acids relative content of natural proteins and NBPs.

	**Natural**	**NBPs**
**% Aliphatics**	33.35	25.25
**% Aromatics**	7.49	14.91
**Aliphatics/Aromatics**	4.45	1.69

### Massive proteins structure prediction environment

**Figure 2:**
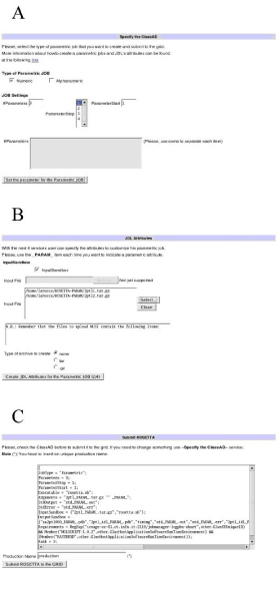
**Genius grid services**. Screenshots of the GENIUS grid portal [13] showing services for the specification of the number of structure predictions to run (A), of the input and output files (B) and for the inspection of the parametric JDL file (C).

To be able to analyse the entire NBPs dataset in a reasonable timeframe, Rosetta *abinitio *has been deployed on the EUChinaGRID grid infrastructure [14] and a user friendly job submission environment has been developed within the GENIUS Grid Portal [12-14]. Figure 2 shows typical GENIUS screenshots of Rosetta parametric job run setup in grid. In particular, using the web interface developed within GENIUS, the user can easily and transparently specify the number of predictions to be made within a single job (Figure 2A), specify the program executable and input files to be uploaded in grid (Figure 2B), inspect the JDL (Job Description Language) file created and submit the job to the grid. This job submission environment, together with the computing power supplied by the EUChinaGRID grid infrastructure, allowed to predict the three-dimensional structure of about one hundred NBPs per day.

**Figure 3:**
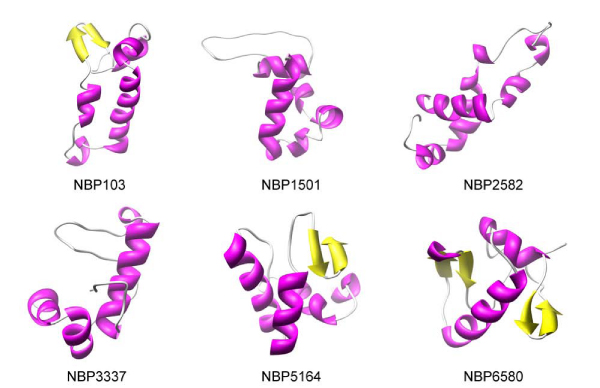
**Sample NBPs predicted three-dimensional structures**. Schematic representation of the three-dimensional structure of randomly chosen NBPs. α helices are coloured in magenta, β strands in yellow.

**Figure 4:**
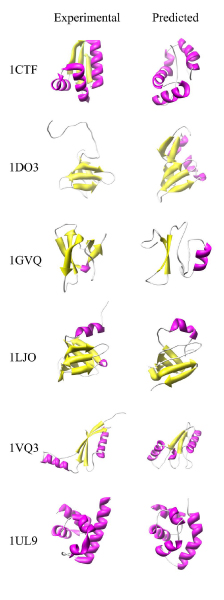
**Sample natural proteins predicted three-dimensional structures**. Schematic representation of the experimental (left panel) and predicted (right panel) three-dimensional structures of randomly chosen natural proteins. α helices are coloured in magenta, β strands in yellow. Protein Data Bank [11] identification codes are indicated on the left for reference.

Figure 3 shows some, randomly chosen, examples of the approx. 2 × 10^4 ^NBPs three-dimensional structures predicted using Rosetta in grid. To provide a measure of the reliability of Rosetta predictions, six natural proteins were also chosen randomly and their three-dimensional structures were predicted using Rosetta. The predicted structures are shown in figure 4 together with the corresponding experimental structures for comparison.

### Comparative structural analysis

Several structure-related parameters (volume, surface, surface/volume ratio, secondary structure content, and surface hydrophobicity) have been computed for the two datasets in order to compare their statistical and structural properties (Table 3).

**Table 3:** Average values^a ^of the structure-related parameters calculated for natural proteins and NBPs.

	**Natural**		**NBPs**	
**Surface (Å**^2^**)**	3920.0	(625.0)	3955.6	(239.8)
**Volume (Å**^3^**)**	8629.0	(1191.6)	9294.2	(359.2)
**Surface/Volume (Å)**	0.46	(0.07)	0.43	(0.03)
**% α helix**	21.4	(17.9)	31.0	(8.8)
**% β strand**	14.4	(12.2)	7.3	(4.3)
**% β turn**	25.6	(13.1)	24.7	(9.9)
**Surface hydrophobicity**	0.36	(0.07)	0.38	(0.05)

^a^Standard deviation values are given in parenthesis.

As a general consideration, the average value of the analysed structural parameters and the corresponding standard deviation values are statistically different between NBPs and natural proteins with a significance level of 0.05. In particular natural proteins are characterised by a higher standard deviation whereas NBPs seem to be narrowly distributed around the experimental average.

**Figure 5:**
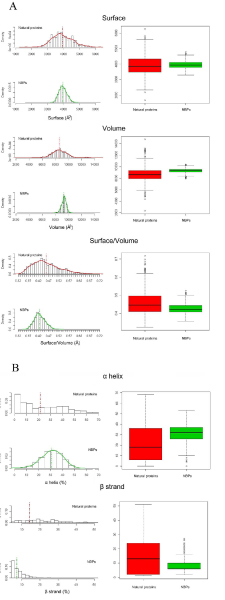
**Structural properties of NBPs and natural proteins**. A) Surface, volume and surface/volume ratio distribution for NBPs and natural proteins; B) Secondary structure content of NBPs and natural proteins. In this and in the following figure, boxplots are shown in the right panels

Despite the differences observed in amino acid composition between the two datasets, the structural analysis of NBPs reveals that these are in most cases characterized by a well ordered structure. In fact, secondary structure content of NBPs appears to be comparable to that of natural proteins, with an average total secondary structure content (including α helix, β strands and β turn) slightly higher than 60% for both datasets (Table 3). However, NBPs appear to be less compact than natural proteins, as evidenced by the higher average volume (9294.0 Å^3 ^and 8630.0 Å^3 ^for NBPs and natural proteins, respectively, Table 3) and lower surface/volume ratio (0.43 Å and 0.46 Å for NBPs and natural proteins, respectively, Table 3) (Figure 5A). Interestingly, NBPs display a significantly higher α helix content, and a corresponding lower β strands content, with respect to natural proteins (Figure 5B), in the absence of any clear correlation between amino acid composition and amino acids propensities for formation of a specific secondary structure.

**Figure 6:**
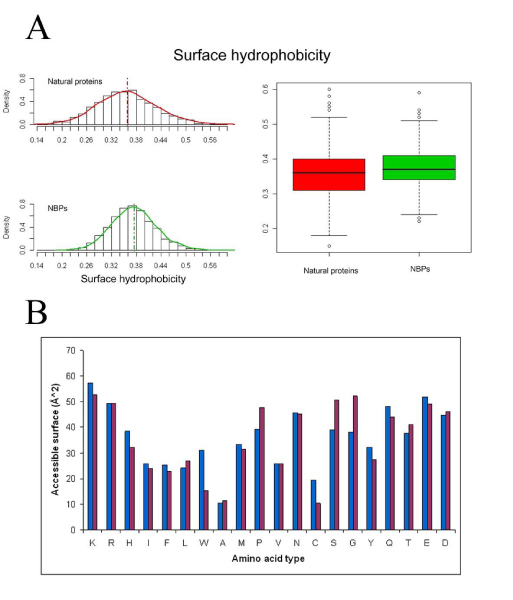
**Surface properties of NBPs and natural proteins**. A) Surface hydrophobicity of NBPs and natural proteins; B) Amino acids solvent accessibility for NBPs (blue) and natural proteins (purple).

Surface hydrophobicity of the two datasets has also been calculated and results to be very similar (Figure 6A), indicating a predicted water solubility of NBPs structures comparable to that of natural proteins. Interestingly, comparison of the amino acid composition of the two datasets with data relative to solvent accessibility of different amino acids types, highlights how aromatic amino acids are more represented in the hydrophobic core of NBPs with respect to natural proteins. As an example, Trp residues are approximately three times more frequent in NBPs with respect to natural proteins (Figure 1A). However, solvent accessible surface of NBPs Trp residues is, on average, only twice that of natural proteins Trp residues (Figure 6B), indicating that a higher proportion of Trp residues is buried within the hydrophobic core of NBPs. Similar considerations apply to Phe and Tyr residues (compare Figures 1A and 6B), leading to the conclusion that aromatic residues contribute to NBPs hydrophobic core formation to a higher degree than in natural proteins.

## Discussion

Meaningful interpretation of the results described in the present work rely heavily on the validity of the structure predictions obtained using Rosetta. However, the Rosetta model has been shown to perform fairly well and even yield near-atomic resolution structures in a number of cases [4]. Results shown in figure 4 for a sample of natural proteins confirm that Rosetta predictions are in most cases fairly accurate in terms of overall fold, secondary structure content and topology. In some cases the agreement between the experimental and predicted structures is even surprising, as is the case of the predicted structure of the protein nusa (indicated in figure 4 with the PDB code 1UL9) which displays an overall backbone r.m.s.d. of only 1.74 Å with respect to the experimentally determined structure.

Analysis of the structural properties of the predicted NBPs structures yielded several interesting and in some cases counterintuitive results. In fact one would expect that in a large population of random amino acid sequences, a large proportion would be "unfoldable" and thus unstructured. Given the assumption of the Rosetta model, our results indicate that this is not the case. Indeed most of the NBPs structures are compact and well ordered, as indicated by the average surface/volume ratio and secondary structure content (Figure 5 and Table 3). Surface polarity is similar to that of natural proteins (Figure 6) suggesting that water solubility is an intrinsic property of random polypeptides. The main differences observed between NBPs and natural proteins are the lower compactness and higher α helix content of NBPs.

The lower compactness observed for NBPs is probably related to their significantly higher aromatic/aliphatic residues ratio with respect to natural proteins (Table 2). In fact, a higher proportion of aromatic residues in NBPs results in a hydrophobic core composition more prone to packing "defects", given the rigid character of aromatic sidechains with respect to branched aliphatic residues such as Leu. Indeed, Leu is largely over represented in natural proteins while the opposite is observed for aromatic residues (Figure 1).

The latter finding has important evolutionary implications. In fact a hydrophobic core made up of branched aliphatic amino acids is probably more tolerant to mutations in that residue substitutions are more easily accommodated by conformational changes of the flexible aliphatic side chains.

Regarding secondary structure content, NBPs display a higher α helix content with respect to natural proteins and a very low β strands content (Figure 5 and Table 3). This could be related to the local nature of the interactions within the α helix. In fact a helical fold can accommodate random sequences by packing together α helical elements interrupted by loops in which bad helix forming residues are located. This is much more difficult in β sheets in which precise pairing of β strands, far away from each other along the amino acid sequence, is required to form a stable structure. From this point of view it can be hypothesized that helical folds are more tolerant to random amino acid sequences. This is a fascinating hypothesis that would be very interesting to test experimentally. In fact in a pre-biotic scenario, in which the first polypeptides were probably characterized by random amino acid sequences, α helix could have emerged early as an intrinsic structural property of polypeptides.

## Conclusion

Results reported in this work highlight how the computational study of "never born proteins", though predictive in nature, can give a useful insight on the basic structural properties of polypeptides and on the specific properties of natural proteins. NBPs appear to be structurally very similar to natural proteins, suggesting that the enormous sequence space of NBPs could indeed be exploited for biotechnological purposes. An important difference between NBPs and natural proteins resides in the different aromatic/aliphatic amino acids content, and in particular in the lower content of aromatic amino acids observed in natural proteins. This information can be very useful in the design of directed evolution and protein engineering studies.

Finally, this study demonstrates that exploitation of grid infrastructures for massive structure prediction projects is feasible, possible applications including genome wide protein structure prediction of bacterial pathogens for target selection and drug design studies.

## Competing interests

The authors declare that they have no competing interests.

## Authors' contributions

GM carried out NBPs structure predictions and analysis, and drafted the manuscript. GE generated the NBPs sequence library and helped in the structures analysis. LV, DS, DDL and IP performed the statistical analysis of the data and helped to draft the manuscript. PLL participated in the design of the study and in drafting the manuscript. FP conceived the study, participated in its design and coordination and helped to draft the manuscript. All authors read and approved the final manuscript.
